# Classification of emotional stress and physical stress using a multispectral based deep feature extraction model

**DOI:** 10.1038/s41598-023-29903-3

**Published:** 2023-02-15

**Authors:** Kan Hong

**Affiliations:** Jiangxi University of Finance and Economics, Nanchang, China

**Keywords:** Engineering, Mathematics and computing, Optics and photonics

## Abstract

A classification model (Stress Classification-Net) of emotional stress and physical stress is proposed, which can extract classification features based on multispectral and tissue blood oxygen saturation (StO_2_) characteristics. Related features are extracted on this basis, and the learning model with frequency domain and signal amplification is proposed for the first time. Given that multispectral imaging signals are time series data, time series StO_2_ is extracted from spectral signals. The proper region of interest (ROI) is obtained by a composite criterion, and the ROI source is determined by the universality and robustness of the signal. The frequency-domain signals of ROI are further obtained by wavelet transform. To fully utilize the frequency-domain characteristics, the multi-neighbor vector of locally aggregated descriptors (MN-VLAD) model is proposed to extract useful features. The acquired time series features are finally put into the long short-term memory (LSTM) model to learn the classification characteristics. Through SC-NET model, the classification signals of emotional stress and physical stress are successfully obtained. Experiments show that the classification result is encouraging, and the accuracy of the proposed algorithm is over 90%.

## Introduction

### Related research

As an important physiological signal, stress has been widely valued by the field. Stress is a cognitive and behavioral experience process composed of psychological stress and physiological stress reaction. Stress can be defined as any type of change that causes physical, emotional, or psychological strain. Stress is the body’s response to anything that requires attention or action. Furthermore, stress can affect people’s physical and mental health, which has long been recognized. According to different stressors, stress can be divided into emotional stress (ES) and physical stress (PS)^1–4^. ES can be triggered by real or perceived external threats. This stress triggers defensive behavioral responses accompanied by appropriate neuroendocrine, autonomic, and respiratory responses. Different pathways in the brain sub serve different components of the response. PS comes from external stimuli, such as excessive exercise and working or driving long hours. Short-term PS gives people a feeling of exhaustion and listlessness, and the most apparent form of PS is through an acute injury^5,6^. The effects of ES and PS on the human body have been widely studied. Excessive ES will harm the health of human’s nervous, musculoskeletal, respiratory, cardiovascular, endocrine, gastrointestinal, reproductive, and other systems. At the same time, the influence of PS on human’s health and specific diseases (symptoms) has been studied over the past decades^7–12^. However, given their different mechanisms, the effects of the two types of stress on the human body will be obviously different. Therefore, they should be classified efficiently for medical diagnosis. Moreover, an effective judgment basis from the two types of stress classification information can be obtained from daily physiological health monitoring. In addition to medical applications, a single stress information can also be used as the initial screening information of security. For example, ES signal can be used as the discrimination signal of security intent detection. However, PS does not have this potential, so it is of practical significance to classify ES and PS in the field of security.

Stress detection and classification has been a research hotspot, starting from contact medical analysis. When a person is under stress, the sympathetic nervous system (SNS) starts to drive the “fight or flight” response. At the same time, as one of the most important reactions of SNS, stress stimulus can induce a complex chemo-electronic analysis procedure (hypothalamic–pituitary–adrenal axis); meanwhile, cortisol level and heart rate (HR) will be driven by this series of reactions, so cortisol level and HR are recognized as stress marker in the field^13–23^. However, medical analysis is a contact method, which is tedious and time-consuming. Therefore, the research direction in the field gradually tends to noncontact imaging methods, even though galvanic skin response, electrocardiography, functional magnetic resonance imaging technologies, and other technologies can obtain more intuitive physiological data^24–35^. The stress detection method was first proposed and developed based on thermal infrared imaging^27,36–43^. This study found that the temperature of facial periorbital region will change obviously under stress, which is accompanied by a series of physiological signals, such as sweating, blood flow velocity, HR, blood perfusion, and breathing. Recently, some researchers have tried to use imaging systems based on RGB sensors or cheaper mobile phones with imaging sensors to identify stress. Various algorithms that use facial features to recognize stress are also encouraging. For example, eye blink rate and eye closure duration caused by sleep deprivation or directed attention are used to recognize PS. In addition, other features such as head pose, head motion (shaking), and yawning in face video are adopted by the field^44–60^.

With the development of artificial intelligence technology, convolutional neural network, residual neural network, dense Convolutional network, long short-term memory (LSTM) and countermeasure neural network are also widely used in the field of computer vision^61–82^, providing a more effective solution for stress feature extraction. Until spectral imaging technology was introduced, directly associating image features with physiological parameters and classifying them has been difficult. Prior to beginning the aforementioned research on spectral imaging, considerable efforts have been made on thermal infrared imaging. Facial thermal imprint signals and HR were successfully correlated, and classified thermal signals of PS and ES were extracted in the forehead region^83^. However, thermal infrared signal is highly unstable. Especially, it is affected by environmental and human body temperature, which will affect the accuracy of algorithms.

In comparison with thermal infrared imaging technology, spectral imaging is less affected by external temperature and environmental factors, so it is a concern in the biomedical field. Spectral imaging technology has developed rapidly, including grating spectroscopy, acousto-optic tunable filter spectroscopy, prism spectroscopy, and chip coating; such technology can be applied in food safety, medical diagnosis, aerospace, and other fields^84–97^. In the past decades, hyperspectral imaging (HSI) technology has been applied to stress recognition for the first time^98–102^, and experimental results are highly encouraging. However, the frame rate of HSI is extremely low, which cannot obtain real-time face data^103^. This drawback further affects the study of real-time stress classification. In this study, real-time facial features are extracted by multispectral imaging system (MSI) for the first time to realize the classification of ES and PS.

### Present research

In this study, a classification model of ES and PS based on facial tissue blood oxygen saturation (StO_2_) signal and frequency-domain deep learning is proposed. Previous studies have extracted StO_2_ signals from the human face using spectral imaging system, and experimental results have proved that facial StO_2_ signals change obviously when the human body is under stress^98–102^. This change is based on the stress response of the human body, but each type of stress has different effects, which will inevitably bring different responses to StO_2_ signal. However, directly correlating the time series StO_2_ signal of a specific region of interest (ROI) with ES or PS is difficult, which brings great challenges for direct classification. Therefore, the current research cannot adopt the method of direct correlation. Thus, the algorithm model of deep learning should be used to mine classification features.

The successful development of algorithm models based on time series data makes it possible to apply deep learning in the field of affective computing. To learn the PS and ES classification signals from StO_2_, this study proposes to extract classification features using frequency-domain information for the first time and focuses on the spatial–frequency component. In the spatial domain, traditional facial features are calculated by geometric structure and image gradient, but the features of StO_2_ signal are not determined by geometric features. However, the use of frequency-domain signal as feature extraction in the field of affective computing has been rare, and combining frequency-based deep learning method and StO_2_ signal to extract stress classification features has not been conducted in relevant research in the field.

The above insights have inspired this study to take the advantages of StO_2_ and spectral signals in the frequency domain and develop a deep learning model for the classification of ES and PS. The proposed Stress Classification-Net (SC-Net) algorithm is based on StO_2_, and the required ROI is obtained systematically through compound constraints. Subsequently, the frequency-domain information is extracted by wavelet analysis, and the corresponding sub-bands are amplified to obtain better classification signals. Finally, the improved multi-neighbor vector of locally aggregated descriptors (MN-VLAD) algorithm is used for feature extraction. The extracted features are then put into the LSTM model for final classification. The main contributions of this study can be summarized as follows.

- A classification method of ES and PS based on MSI technology is proposed for the first time. To the best of the authors’ knowledge, this study is the first attempt in the field of affective computing to classify PS and ES by using real-time spectral signals and time series StO_2_ features.
- A feature extraction algorithm model based on frequency domain is proposed for the first time. The algorithm can learn the classification features of different frequency domains based on wavelet transform and highlight their respective characteristics by signal amplification.
- A feature extraction method based on MN-VLAD algorithm is proposed and applied to frequency domain to improve the performance of the algorithm.

The remainder of this paper is organized as follows. Section “Acquisition of experimental data” describes the experimental settings during MSI data acquisition, and the algorithm model is demonstrated in Section “Algorithm model”. Sections “Experimental results and analysis” and “Discussion and conclusion” present the analysis of the results and the conclusions, respectively.

## Acquisition of experimental data

Most of the participants in the experiment were recruited from the media. The 260 healthy volunteers who participated in the experiment came from different races and colors (i.e. Caucasian, Indian, Chinese, Malaysian, and South African), among which 55% were male and 45% were female. They ranged in age from 20 to 65, with an average age of 37.7 years old (SD 17.75). All the participants provided written informed consent. Of all the 260 participants, 50% of the data were used for algorithm training, and the remaining 50% were used for testing. All the participants were asked to wear a chest strap heart monitor (Garmin GARMINACTIVE S) and a finger probe (Miroxi Oximeter—Pulse) to test their HR. Saliva sample were collected in a 5-min interval using Salivette. The participants were also asked to insert the saliva collection swabs in their mouths. The sample was stored in a freezer until analysis by time-resolved fluorescence immunoassay.

In this study, the Trier Social Stress Test (TSST) was used to trigger acute ES^105–107^. Stressors in standard TSST are usually represented by public speaking in the form of interview and mental arithmetic, which makes participants have the characteristics of social-evaluative threat. This process is the most important to trigger cortisol release under stress. For each participant, mental arithmetic was performed within a strict time scale of 4–5 s, which could introduce rising psychological stress by increasing the difficulty of the problem. After mental arithmetic, all the participants were asked to give a public speech in the form of job interview or project description and provide answers in front of judges or interviewees. To supplement the previous emotional stressors, recognition and memory task was used to improve the experimental effect, and the process of these tasks was carefully designed. In the experiment, the participants were given a memory test task, that is, the participants needed to remember a group of two images appearing in the same slide simultaneously. During the learning process, a series of image combinations would flash, and each image combination would remain on the monitor for a period of 2 s. The learning process consisted of about 6–10 slides, and time was short. After learning, the participants were asked to identify which two of the five images that appeared in the test slide were the group shown earlier. The answer time was strictly limited to 5 s. Based on the above process, the emotion stress stimuli last around 45 min. Through the aforementioned experiments, the ES response of the participants could be stimulated to varying degrees. Conversely, the experiment of PS was relatively simple, and the participants mainly stimulated PS by running.

**Figure 1:**
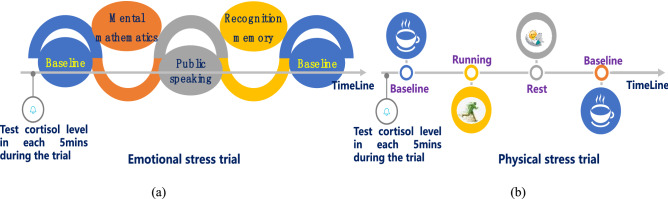
Process of (**a**) ES experiment and (**b**) PS experiment.

The PS experiments were completed as follows. The participants were required to wear HR testing equipment (Garmin and Miroxi types). Then, they were then assigned to a room with better lighting conditions and were asked to sit down comfortably. Subsequently, the participants were given 5 min to adapt to the new environment. Then, they were asked to run as hard as possible for 5 min, and they were given a 20-min rest after running to return to the baseline state. At the same time, the MSI system was constantly running to record real-time data. By contrast, the ES experiment was slightly more complicated. A series of TSST stressor was implemented after the participants adapt to the new environment and wear HR test equipment, mainly applying mental mathematics and public speaking to the participants in the form of interview and recognition memory task. Thereafter, a recovery time of about 45 min would allow the participants to return to the baseline state. The process of the experiment is shown in Fig. 1.

In this study, the MSI covered visible and near-infrared bands, ranging from 450 to 800 nm. The whole system consisted of a Tamron lens, a Brimrose acoustic optic tunable filter imaging spectrograph, and a computer. The area CCD array detector of the camera (BM-141GE, Japan) contained 1392 (h) × 1040 (v) active pixels with a spectral resolution of 2 nm. The frame rate of MSI was set to 30 Hz, and Matlab (Matlab R2019a) and Baotfis (Baotfis 2.0) software were used for data acquisition and algorithm analysis. The author confirms that the use of human face samples is performed in accordance with relevant regulations of Jiangxi University of Finance and Economics. Moreover, all experimental protocols were approved by the research committee of Jiangxi University of Finance and Economics. All methods were performed in accordance with the guidelines and regulations of Jiangxi University of Finance and Economics. The informed consent was obtained from all subjects for publication of their experimental data in an online open-access publication.

## Algorithm model

The main step of the proposed algorithm begins with the extraction of facial StO_2_ signal. Given the high imaging frame rate and fast reaction, real-time facial spectral signals can be extracted by using MSI. Before the feature extraction, corner point detection and optical flow method are employed to eliminate the effect of the participants’ tremble. Through these facial signals, time series StO_2_ signals can be inverted. The selection of the ROI in many previous studies is simple and random, which may lead to the decline of the reliability of the data source. Under PS or ES, the StO_2_ signal of the ROI will change. Thus, in selecting the ROI, the ROI must be able to keep the same change trend among different people as much as possible. In this manner, the StO_2_ signal of the ROI can have certain universality, rather than the characteristics of a single person or a few people. Furthermore, the selection of the ROI must be representative and judged based on the signal strength of the StO_2_ signal. To this end, signal robustness is also an important criterion for selecting ROI. For this purpose, the ROI selection is quantized by a composite criterion, and the ROI signal source is determined by the universality and robustness of the signal. Subsequently, the frequency-domain information is extracted by wavelet analysis, and the corresponding sub-bands are amplified to obtain better classified signals. To fully utilize the frequency-domain characteristics, the MN-VLAD algorithm is proposed to extract useful features. Finally, the extracted features are put into the LSTM model to learn the classification characteristics to complete the classification of PS and ES.

**Figure 2:**
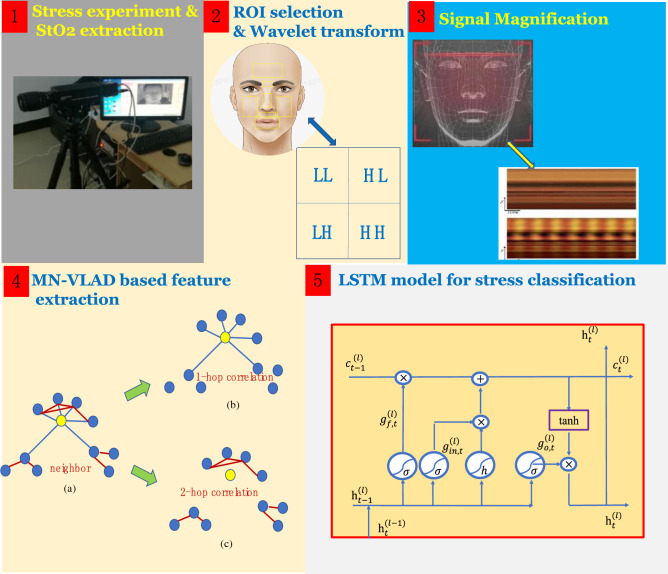
Process of SC-Net. The whole process starts from the experiment. After obtaining the StO2 signal, we extract the relevant signal of interest through ROI selection. Then, features are extracted by signal amplification and MN-VLAD based model. Finally, all features are put into LSTM model to complete stress classification.

The process of the whole SC-Net algorithm is shown in Fig. 2. The SC-NET starts from the stress experiment. After obtaining the StO_2_ signal, we extract the relevant signal of interest through ROI selection. Then, features are extracted by signal amplification and MN-VLAD based model. Finally, all features are put into LSTM model to complete stress classification.

### StO_2_ extraction

The StO_2_ extraction algorithm is based on MSI technology and the light absorption effect of facial skin. The four main chromophores of facial skin absorption spectrum are deoxyhemoglobin (Hb), oxyhemoglobin (HbO_2_), scattering effect, and melanin. Given that the light absorption of each chromophore varies in different wavelengths, the commonly used StO_2_ extraction algorithms are mostly based on the Beer–Lambert (BL) model^101,102,104–109^. The BL model describes the relationship between the light absorption and the thickness of the absorbing medium, which can be expressed as follows:

1 $$ A = \varepsilon_{{HbO_{2} }} C_{{effHbO_{2} }} + \varepsilon_{Hb} C_{effHb} + \varepsilon_{melanin} C_{effmelanin} + G^{\prime}, $$

where *A* is the rate of absorption; *ε* is the molar absorptivity of HbO_2_, Hb, and melanin; and *c* is the effective concentration (*10*^*−3*^* mol/cm*^*2*^). *G′* represents all parameters, such as skin mirror reflection and regression error, which are not related to the absorption rate of tissues; these noises are not marginal but can be readily solved. The effective concentrations of C_effHbO2_ and C_effHb_ estimated from Eq. (1) are powerful indicators of the actual molar concentrations of HbO_2_ and Hb. By solving Eq. (1), the values of C_effHbO2_ and C_effHb_ can be inferred to be approximately linear with the actual concentrations of HbO_2_ and Hb^108^. Thus, the *A* and molar absorptivity value sets (HbO_2_, Hb, and melanin) of the four wavelength datasets can determine C_effHbO2_ and C_effHb_. Therefore, the noise *G′* can be readily eliminated. Furthermore, the effective concentrations of Hb, HbO_2_, StO_2_, and errors can be solved by the simultaneous decomposition of data of different bands. In the aspect of band selection, the extinction coefficient correlation of Hb, HbO_2_, and melanin in the human skin is considered completely. Generally, the extinction coefficient of melanin decreases rapidly with the increase of band^110,112^. The weight of light absorption is low when the light infrared range is near. Given the signal source, the algorithm cannot use the ultraviolet band to reduce the weight of melanin, although Hb and HbO_2_ have multiple absorption peaks in the ultraviolet band. At 600 nm of the extinction coefficient of Hb and HbO_2_, a cliff-like decline can be found, whereas melanin also has the same order of magnitude of decline. Therefore, in this study, the corresponding StO_2_ signals are extracted from the MSI data in the four bands, and the band range is controlled in the 500–600 nm range (i.e. 540, 556, 560, and 576 nm).

### ROI selection

The selection of the ROI must be representative and judged based on the signal strength of the StO_2_ signal. The multisubject correlation method for ROI selection has been proved successful in previous research^83,102^. Although multisubject correlation-based method can be used to judge whether the signal changes are universal, the strength is impossible to assess. Therefore, this study proposes the selection of the ROI based on compound conditions.

#### Multisubject correlation

**Figure 3:**
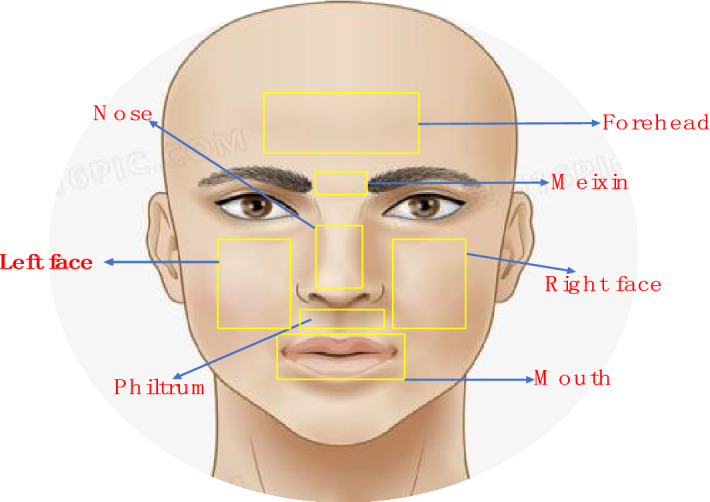
Example position for the selected ROI (Note: Meixin is the eyebrow center (middle eyebrow region)). The ROI of the face is defined as different objects as possible ROI, such as forehead, nose, left face, right face, mouth, meixin and philtrum.

In this study, the ROI is preliminarily selected by multisubject correlation. The ROI of the face is defined as different objects as possible ROI, such as forehead, nose, left face, right face, mouth, meixin (middle eyebrow region), and philtrum (Fig. 3). Since the experimental environment is indoor, the StO_2_ image we obtained can be “standardized” by image registration. Therefore, we first obtain 68 key points of the face^111^. Through the obtained key point matrix and geometric transformation, all original key point matrices can be mapped to the same size. Therefore, a fourth-order polynomial transformation matrix can be achieved together with registered StO_2_ image. To this end, all StO_2_ images can be standardized. The ROIs can be thereby obtained and standardized. As long as the location of the initial ROI is defined, the ROI of all other StO_2_ images can be further obtained readily.

The StO_2_ signals in ROIs are expected to be sensitive to stress and have good cross-correlation. However, this sensitivity must be built on all participants. On this basis, the ROI can be assessed by correlation. The most commonly used correlation discrimination algorithm is Pearson coefficient; however, different from Pearson coefficient, this study has more than two objects to build a correlation (the participants are considerably larger than two). Pearson coefficient can only calculate the correlation between two sets of data; thus, this study uses a multivariant correlation method^113^ for the analysis of interrelations between facial StO_2_ signal. This method is based on the zero-lag correlation matrix and random matrix theory technology, which can effectively detect and characterize spatial–temporal correlation patterns. Particularly, the correlation of multiple objects can be constructed. When the number of objects is N ≥ 2, the multivariant correlation method can evaluate the correlation of data from multiple channels, which is needed in this study.

An equal-time correlation matrix is initially set. A measured StO_2_ multi-ROI time series signal is set to S*T*_*i*_*(t) (i* = *1, …, N)*, and the equal-time correlation matrix *CR*_*ET*_ is constructed by normalizing the StO_2_ signal, as shown as follows:

2 $$\widetilde{{ ST}_{i}}\left(t\right)=\frac{{ST}_{i}\left(t\right)-\overline{{ST }_{t}\left(t\right)}}{{\sigma }_{i}\left(t\right)},$$

where ${\sigma }_{i}$ denotes the standard deviation, and $\overline{{ST }_{t}}$ is the mean. By using Pearson correlation coefficient to analyze each parameter, the equal-time correlation matrix C*R*_*ET*_ is set as follows:

3 $${CR}_{ETij}\left(t\right)=\frac{1}{T}\sum_{t=1}^{T}\widetilde{{ST}_{i}}\left(t\right)\widetilde{{ST}_{j}}\left(t\right)={\langle \widetilde{{ST}_{i}}\widetilde{{ST}_{j}}\rangle }_{t.}$$

In this case, all calculations are based on the time series t ∈ [1, T], from which the equal-time correlation matrix can be rewritten as

4 $${CR}_{ET}=\frac{1}{T} \widetilde{ST }{\widetilde{ST}}^{^{\prime}},$$

where ${\widetilde{ST}}^{^{\prime}}$ is the transposition of $\widetilde{ST}$. The correlation structure *ST*_*i*_ of different participants is contained in bivariate measures and represented by *N(N − 1)/2* independent matrix coefficient *CR*_*ET*_. Matrix *CR*_*ET*_ can interpret the cross-correlation between specific participant data and other participant data, and this method is simple in calculation and can provide a considerably direct interpretation. The eigenvalue and eigenmatrix of matrix *CR*_*ET*_ produce the joint probability distribution of basic processes, and the eigenvalue can provide the similarity. For an infinitely long time series, if the StO_2_ signals of all the participants in a certain ROI are not correlated at all, then the nondiagonal element of the matrix *CR*_*ET*_ will be equal to 0. On the contrary, if the signals are completely correlated, then the *CR*_*ET*_ element of the matrix will be equal to 1. Therefore, the correlation between data depends on the maximum eigenvalue of *CR*_*ET*_. In this study, the *ST*_*i*_ (ROI StO_2_) signal of all finitely long random time series is normalized. After normalization, the eigenvalue distribution of the correlation matrix of *ST*_*i*_ can be written as follows:

5 $$E\left(\lambda \right)=\frac{U}{2\pi }\frac{\sqrt{({\lambda }_{+}-\lambda )(\lambda -{\lambda }_{-})}}{\lambda },$$

where $\lambda \epsilon [{\lambda }_{-}, {\lambda }_{+}]$, *U* is a parameter that always equal to *T/N*; thus, ${\lambda }_{-}$ and ${\uplambda }_{+}$ can be obtained as follows:

6 $${\lambda }_{\pm }(U)=1+\frac{1}{U}\pm \frac{2}{\sqrt{U}}$$

In the StO_2_ signals of the participants, the correlation matrix CR_*ET*_ represents the correlation of various StO_2_ data. Only the maximum eigenvalue should be calculated and compared with ${\uplambda }_{+}$. Given that ${\uplambda }_{+}$ can evaluate maximum random correlations, if the maximum eigenvalue is larger than ${\uplambda }_{+}$, then there exists certain similarity and correlation; otherwise, no exists correlation between ROI signal data. Therefore, the ROI StO_2_ signals of different participants are put into the model to analyze whether there exists a certain correlation one by one to determine whether the correlation of the ROI is universal. Therefore, the first ROI selection parameter is obtained and defined as ${{{\uplambda }_{\mathrm{MSC}}=\uplambda }_{\mathrm{max}}/\uplambda }_{+}$. According to ${\uplambda }_{\mathrm{MSC}}$, the multiobject correlation and universality of the ROI can be determined.

#### Compound conditions for ROI selection

The second parameter for ROI selection is based on the signal strength of the StO_2_. The StO_2_ signal of the ROI is expected to have strong changes under stress. The traditional method compares the time series signals according to different positions and explores the timing signals with strong reaction among different ROIs. However, this matching process is complex and time-consuming. To this end, wavelet variations are used to circumvent this process.

The dataset is initially defined, and the facial ROI time series signal is ${f}_{ROI}\left(t\right)={({f}_{ROI}^{{M}_{1}}\left(t\right), {{f}_{ROI}^{{M}_{2}}\left(t\right),{ f}_{ROI}^{{M}_{3}}\left(t\right), {f}_{ROI}^{{M}_{4}}\left(t\right), f}_{ROI}^{S}\left(t\right))}^{T}$, which describes the signal changes of the ROI time series StO_2_ and spectral signals somewhere in the face. Particularly, ${f}_{ROI}^{{M}_{i}}\left(t\right)$ and ${f}_{ROI}^{S}\left(t\right)\in {R}^{2}$ represent the spectral time sequence signal and the StO_2_ time sequence signal of a certain band in the ROI, respectively. Here, StO_2_ is obtained by 4-band inversion, which results in 4-band data corresponding to StO_2_ signal. Thus, the dataset consists of 5 channel data.

The mean and variance of the related image signals can also constitute the feature vector (FV) of a certain ROI, which yields the FV as follows:

7 $$FV={\left({\mu }_{1}^{{M}_{1}}, {\sigma }_{1}^{{M}_{1}},{\mu }_{1}^{{M}_{2}}, {\sigma }_{1}^{{M}_{2}},\dots {\mu }_{1}^{s}, {\sigma }_{1}^{s},{\mu }_{2}^{{M}_{1}}, {\sigma }_{2}^{{M}_{1}},\dots {\mu }_{i}^{s}, {\sigma }_{i}^{s},\right)}^{T},$$

where $\mu$ is the mean; $\sigma$ is the variance; *i* is the frame number; *M*_*1*_*, …, M*_*4*_ is the number of band channels; and *S* refers to StO_2_ channels. Thus, the basic signal of the ROI is obtained, and its response to stress is assessed by this basic signal. This study anticipates that it only needs to assess whether the ROI is suitable according to the change of the ROI signal in a certain time period, that the ROI signal should be matched with the whole image signal in a certain time period. Within this time period, a large part of the signals may be under strong stress response, and only a small part of the signals may be under stress response. Therefore, the proposed method needs to avoid time parameters; otherwise, finding sensitive time periods will be time-consuming. To address this problem, wavelet transform is introduced, and the wavelet transform equation of ${FV}_{ROI}$ is as follows:

8 $$W\left(FV,t\right)=\frac{1}{\sqrt{a}}{\int }_{-\infty }^{\infty }FV\left(t\right)*\psi\, \left(\frac{t-b}{a}\right)dt,$$

where $\psi$ is the wavelet basis, *a* shows the scale factor, and *b* refers to the translation factor. A time period [$0$,* P*] from which the wavelet transform can be described is assumed as follows:

9 $$W\left(FV,t\right)=\frac{1}{\sqrt{a}}{\int }_{0}^{P}FV\left(t\right)*\psi \left(\frac{t-b}{a}\right)dt.$$

Equation (9) can actually be regarded as the inner product over [$0$, P] time period. Thus, the entire wavelet transform can be described as:

10 $$W\left(FV,t\right)=\frac{1}{\sqrt{a}}\langle FV\left(t\right),\psi \left(\frac{t-b}{a}\right)\rangle $$

Given that the wavelet transform in this study is continuous in time and frequency domains, signal space can be regarded as an extension of a series of basic functions. The aim here is to find a suitable ROI, and only the general signal trend should be determined. Then, the original signal ${FV}^{j}\left(t\right)=\sum_{l\in z}{a}_{l}\phi ({2}^{j}t-l)$ with step function approximation can be obtained. This process samples the signal at *t*=$0,\frac{1}{{2}^{j}},\dots$ to obtain ${a}_{l}=FV(l/{2}^{j})$, where ${FV}^{j}$ is a step function. If detailed features should be obtained, then *j* must make ${2}^{-j}$ sufficiently small, although it is unnecessary in this study. Therefore, the inner product of two ROI signals can be calculated as follows:

11 $$\langle {FV}_{1},{FV}_{2}\rangle =\sum_{l\in z}{\int }_{0}^{P}{a}_{l1}{\phi }_{1}{({2}^{j}t-l)a}_{l2}{\phi }_{2}({2}^{j}t-l)dt={{a}_{l1}}^{T}\omega \left(t\right){a}_{l2},$$

where $\omega \left(t\right)=\langle {\phi }_{1}{({2}^{j}t-l)a}_{l2},{\phi }_{2}({2}^{j}t-l)\rangle$. At the same time, given that only the general content of the signal should be obtained, only the first component (cosine basis) of the Fourier series of the signal should be calculated. The approximation of *FV*_*1*_ in the time period [$0$, P] can be obtained as follows:

12 $${FV}_{1}=\langle {a}_{l1}, {\phi }_{1}({2}^{j}t-l)\rangle \approx \sum_{l\in z}{{a}_{l1}}^{^{\prime}},$$

where ${{a}_{l1}}^{^{\prime}}$ is the correlation coefficient. The approximate representation of the correlation signal in a fixed time period can be obtained, and the correlation of the signal can also be reflected by the calculation of inner product. Moreover, this process does not need to obtain the information of a specific time point, and it does not need to measure parameters by time. In this manner, the information of correlation coefficient can be directly obtained to find the correlation. By calculating the inner product, the correlation between the known strong stress response signal and the unknown ROI signal can be easily obtained. After normalization, the correlation parameter $\delta$ is defined, and strong and weak ROI signals can be classified by simple Euclidean distance.

After obtaining ${\uplambda }_{\mathrm{MSC}}$ and $\delta$ and considering them comprehensively, the ROI selection formula is defined as follows:

13 $${\varepsilon =m\lambda }_{MSC}+n\delta ,$$

where *m* and *n* are the relevant weights. Thus, the preparation of the ROI selection method is completed, and the feature extraction neural network based on frequency and spatial domains is then constructed in the next step.

### Signal magnification

After obtaining the time series signals of StO_2_ in the ROI, the corresponding classification features should be extracted using deep learning model. The original image signal is initially decomposed by wavelet transform to obtain different frequency domains, and the signal is then amplified to find classification information.

Under wavelet decomposition, the image is decomposed into high- and low-frequency signals. The Mexican hat is used as a wavelet base to decompose the image signal. After obtaining the wavelet decomposed image, the obtained subblocks are amplified. Eulerian magnification (EM) has been used for signal magnification^44,83,101,102^. For practical application, signal amplification has been troubled by noise, especially in the process of amplifying high-frequency signals. Therefore, the signal amplification process in this study addresses this noise better.

In view of the influence of noise on the algorithm, an in-depth processing of noise in this signal amplification is performed. The facial ROI signal is defined as $f(x,t)$, where *x* is the intensity of the ROI at time* t*. The purpose of amplifying signals is to highlight the classified signals of ES and PS. The ROI signals of different subblocks are amplified in EM algorithm. By taking StO_2_ signal as an example, directly assuming that the ROI signal can be roughly expressed by first-order Taylor expansion, and defining the Taylor expression of the ROI signal at time *t* as *f(x* + *δ(t))*, the StO_2_ image can be expressed as

14 $$\mathrm{St}\left(x,t\right)\approx f\left(x\right)+\delta \left(t\right)\frac{\partial f\left(x\right)}{\partial x},$$

where *x* is the intensity of the ROI at time *t*, and *δ(t)* is the displacement equation of the signal. Thus, *St(x, t)* = *f(x* + *δ(t))*, and *St(x, 0)* = *f(x)*. The temporal band-pass filter of *f(x, t)*, that is, *B(x, t)*, is arranged. The displacement signal *δ(t)* is within the band pass of the temporal band-pass filter. Then,

15 $$B\left(x,t\right)=\delta \left(t\right)\frac{\partial f\left(x\right)}{\partial x}.$$

Parameter *a* can be used to amplify the signal and is substituted into the following equation:

16 $${St}_{amplified}\left(x,t\right)\approx St\left(x,t\right)+aB\left(x,t\right) \approx f\left(x\right)+\left(1+a\right)\delta \left(t\right)\frac{\partial f\left(x\right)}{\partial x},$$

17 $${St}_{amplified}\left(x,t\right)\approx f\left(x+\left(1+a\right)\delta \left(t\right)\right).$$

To reduce the influence of noise on the signal, the signal to be amplified is redefined as

18 $$St\left(x,t\right)=St\left(x,0\right)+\left(1+a\right){St}_{t}\left(x,t\right).$$

Here, the above equation is deduced as follows by Taylor expansion:

19 $${St}_{amplified}\left(x,t\right)\approx St\left(x,0\right)+\left(1+a\right)\delta \left(t\right){St}_{x}+\frac{1}{2}{\left(1+a\right)}^{2}{\delta }^{2}\left(t\right){St}_{xx},$$

where ${St}_{x}(x,t)=\partial St(x,t)/\partial x$, and the following can be further obtained:

20 $${St}_{amplified}\left(x,t\right)\approx St\left(x,0\right)+\left(1+a\right){St}_{t}-\frac{1}{2}\left(1+a\right){\delta }^{2}\left(t\right){St}_{xx}{St}_{x}+\frac{1}{2}{\left(1+a\right)}^{2}{\delta }^{2}\left(t\right){St}_{xx}.$$

Given that the Eulerian simulation is based on the Taylor expansion, the Eulerian error can be calculated from it, as shown as follows:

21 $$\omega \approx \left|\frac{1}{2}\left(1+a\right){\delta }^{2}\left(t\right){I}_{xx}-\frac{1}{2}{\left(1+a\right)}^{2}{\delta }^{2}\left(t\right){I}_{xx}{I}_{x}\right|.$$

The error here is actually obtained under the condition of no noise, whereas the signal needs to be further decomposed under the condition of noise. The noisy signal is thus defined as

22 $${\widehat{St}}_{noisied}\left(x,t\right)=St\left(x,t\right)+\tau \left(x,t\right).$$

Here, the noise is defined as $\tau (x,t)$, from which the amplified signal with noise can be defined as

23 $${St}_{amplified\,With\,Noise}={St}_{amplified}\left(x,t\right)+\left(1+a\right){\tau }_{t}+\tau .$$

and the Eulerian error, as a function of noise, can be derived as

24 $${\omega }^{^{\prime}}\approx \left|\left(1+a\right){\tau }_{t}+\frac{1}{2}\left(1+a\right){\delta }^{2}\left(t\right){I}_{xx}-\frac{1}{2}{\left(1+a\right)}^{2}{\delta }^{2}\left(t\right){I}_{xx}{I}_{x}+\tau \right|.$$

Therefore, the signal amplification method with noise processing is obtained. At this time, the setting of amplification interval in signal space becomes an important part of the model. For the amplification factor *a* of the StO_2_ signal, 20 times, which is generally conservative, is used. The amplification frequency interval of the signal needs to refer to the estimated stress marker of human physiology. According to experience, the magnification interval is set between [1.2, 2] Hz. In this manner, the signal can be amplified to an appropriate interval to increase the difference between the PS state and the ES state.

### Feature extraction using MN-VLAD

After obtaining the amplified signal, the corresponding features are further extracted. Although the amplified signal can highlight the signal of interest, the MN-VLAD model is proposed as the feature extraction model to extract the classification features systematically. This study aims to determine the invariant functional representation of spectral and StO_2_ signals by the change of the two-dimensional image signal. VLAD is the abbreviation of vector of locally aggregated descriptors, and its core idea is aggregated. VLAD algorithm is based on SIFT and other traditional manual features, and $\mathrm{R}=[{\mathrm{r}}_{1},{\mathrm{r}}_{2},{\mathrm{r}}_{3},\dots {\mathrm{r}}_{N}]\in {\mathrm{R}}^{D\times N}$ is the local feature matrix of the image, where *D* is the dimension of local features and *N* is the number of local features. First, local features are clustered by K-means algorithm, and *M* visual dictionaries are obtained. Then, each local feature is quantized to the nearest neighbor dictionary $\mathrm{Q}=[{\mathrm{q}}_{1},{q}_{2},{q}_{3},\dots {q}_{M}]\in {\mathrm{R}}^{D\times \mathrm{M}}$, and the residual between it and the nearest neighbor dictionary is calculated. The residual on the dictionary Q is calculated as follows:

25 $${v}_{i}=\sum_{r\in R}(r-{q}_{i}),$$

where ${q}_{i}\in Q$, ${q}_{i}$ is the nearest neighbor dictionaries of local feature; and ${v}_{i}$ is the cumulative sum of all local features of the nearest neighbor dictionary belonging to ${r}_{i}$ and the residuals of ${r}_{i}$ in an image. The VLAD feature descriptor vector ${V=[{v}_{1, }{v}_{2, }{v}_{3, }\dots {v}_{M, }]\in {\mathrm{R}}^{D\times \mathrm{M}}}$ is obtained by aggregating all the residual vectors in the visual dictionary.

Existing VLAD algorithms only calculate the residual of local features and nearest neighbor visual dictionaries, but some local features may have similar or even the same distance from two or more nearest neighbor visual dictionaries. However, when coding features, only considering the residual of nearest neighbor dictionaries will result in losing more information. Therefore, the MN-VLAD method is proposed to replace the nearest neighbor assignment with multiple nearest neighbor assignments, and calculate the residuals between local features with good correlation and multiple nearest neighbor visual dictionaries to improve the expression ability of VLAD feature descriptors.

**Figure 4:**
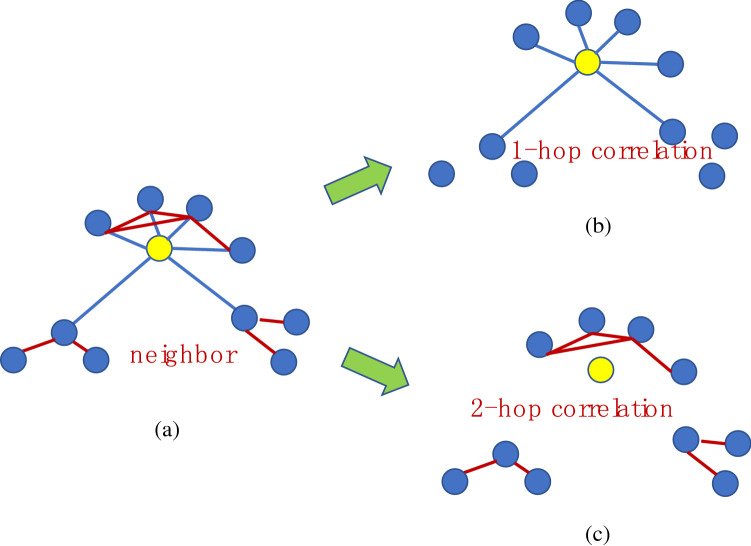
Five-layer MSM structures. The 1-hop and 2-hop neighbors of a feature are dotted in (**a**), and the 1-hop and 2-hop associations in (**c**) are given in (**b**) under the same spatial position, such that the correlation of all features can be fully connected. In the figure, the blue line is a 1-hop connection, and the red line is a 2-hop connection.

To calculate the residuals of adjacent dictionaries, which adjacent points can better reflect the relationship between local features and adjacent dictionary distances should be considered. Then, the correlation between feature points and adjacent dictionaries needs to be taken into account. To this end, a multidimensional spatial relationship map (MSM) in the dictionary is initially defined^83,101,102^. The MSM is designed into five layers, and the adjacent points are then set based on the cluster center KNNs in the MSM; its high-order neighbors are used as the scanning points for MSM. The high-order neighbors are defined up to 2-hop of local features, and 2-hop neighbor is established. The 1-hop node neighbor is defined as 6, and the 2-hop neighbor is defined as 2. The spatial relation template structure is shown in Fig. 4. These local feature neighbors will provide more data infrastructure and feature structure. The 1-hop and 2-hop neighbors of a feature in the first layer of the dictionary are shown in Fig. 4a. The blue line represents a 1-hop connection, whereas the red line illustrates a 2-hop connection. Graphs (b) and (c) show the 1-hop and 2-hop associations under the same spatial position, respectively. When the neighbor points are selected to find a correlation, they are not completely placed in a certain layer, but they are put in all layers in a balanced manner, which is important because the correlation of all features must be fully connected. Therefore, 1-hop and 2-hop correlations will construct the “distance” equation.

Then, the local feature and the distance equation of the neighbor dictionary are calculated, and the correlation on the 1-hop and 2-hop neighbors are indirectly reflected. The correlation matrix of the $\overrightarrow{CLX}$ objective equation can be written as follows:

26 $$\mathrm{min}{\Vert 1-a\overline{{CLX }_{1-hop}}-b\overline{{CLX }_{2-hop}}-\overline{{CLX }_{dictionary}}\Vert }^{2},$$

where integers *a* and *b* are set to the ambiguity. If the integer constraint of ambiguity *a* is ignored, then solving the minimum value can be regarded as a standard least square estimation problem. This solution is commonly referred to as a floating solution, and its estimated value and the associated covariance matrix *X* can be expressed as:

27 $$\left[\begin{array}{c}\widehat{\mathrm{a}}\\ \widehat{\mathrm{b}}\end{array}\right]\left[\begin{array}{c}{\mathrm{X}}_{\widehat{\mathrm{a}}} {\mathrm{X}}_{\widehat{\mathrm{a}}\widehat{\mathrm{b}}}\\ {\mathrm{X}}_{\widehat{\mathrm{b}}\widehat{\mathrm{a }}}{\mathrm{X}}_{\widehat{\mathrm{b}}}\end{array}\right].$$

By using the floating ambiguity solution and its covariance matrix, the integer ambiguity solution $\acute{\text{a}}$ can be calculated as follows:

28 $$\underset{a}{\mathrm{min}}{\left(\widehat{\mathrm{a}}-\mathrm{a}\right)}^{\mathrm{T}}{\mathrm{X}}_{\widehat{\mathrm{a}}}^{-1}(\widehat{\mathrm{a}}-\mathrm{a}).$$

At the same time, the estimation accuracy of the equation can be further improved by using the integer characteristic of ambiguity, as shown as follows:

29 $$\acute{\text{b}} = {\hat{\text{b}}} - {\text{X}}_{{{\hat{\text{b}}}\widehat{{\text{a }}}}} {\text{X}}_{{{\hat{\text{a}}}}}^{ - 1} \left( {{\hat{\text{a}}} - \acute{\text{a}} } \right), $$

30 $${\text{X}}_{{{\acute{\text{b}}}}}  = {\text{X}} - {\text{X}}_{{{\hat{\text{b}}}\widehat{{\text{a }}}}} {\text{X}}_{{{\hat{\text{a}}}}}^{ - 1} {\text{X}}_{{{\hat{\text{a}}}\widehat{{\text{b}}}}} , $$

where $\acute{\text{b}}$ is the fixed solution of the objective equation estimation. The accuracy of the fixed solution of the objective equation estimation is improved compared with the floating solution, and $\acute{\text{a}}$ is the fixed solution of the integer ambiguity. The iteration of the above steps is the process of finding the minimum solution to the objective equation, that is, the optimization process of finding the maximum correlation. The optimal correlation point obtained by this equation is to finally determine the proximity visual dictionary with better correlation with local features. To improve the expressive ability of VLAD feature descriptor, multi-nearest neighbor assignment is used instead of nearest neighbor assignment, and the residual error between local feature and multiple nearest neighbor visual dictionaries is calculated.

### Temporal feature learning with LSTM

**Figure 5:**
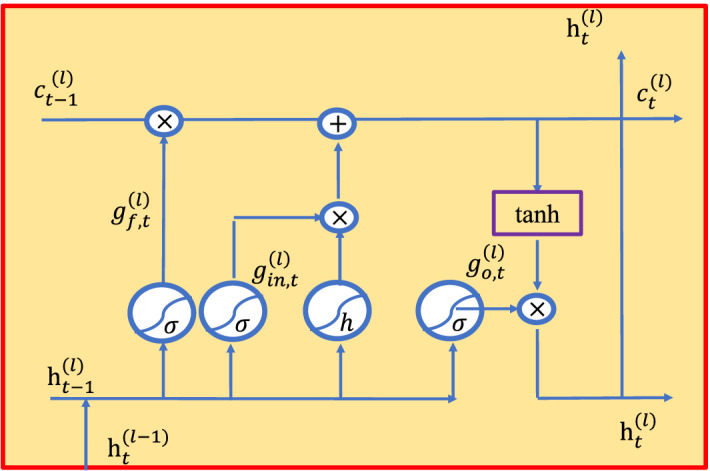
Process of LSTM model. As shown in the figure, ${g}_{in,t}^{(l)}$ denotes the input gate; ${g}_{f,t}^{(l)}$ represents the forget gate; ${g}_{o,t}^{(l)}$ is the output gate; ${c}_{t}^{(l)}$ is the memory cell; and ${h}_{t}^{(l)}$ is the output of the *l*th LSTM layer with a given *t*th input^114^.

After obtaining features, the features are further identified by deep learning to complete the PS and ES classification. The LSTM algorithm is used in model facial dynamics of the variable length feature sequences. LSTM has a high classification accuracy, excellent distributed storage and learning ability, and the function of associative memory^114^. Therefore, the acquired time series features are finally put into the LSTM algorithm. Figure 5 illustrates the flowchart of LSTM model. The LSTM layer illustrated in SC-Net (Fig. 5) operates as follows^114^.

31 $${g}_{in,t}^{(l)}=sigm({W}_{in}^{\left(l\right)}\left[{h}_{t-1}^{\left(l\right)},{h}_{t}^{\left(l-1\right)}\right]+{b}_{in}^{\left(l\right)})$$

32 $${g}_{f,t}^{(l)}=sigm({W}_{f}^{\left(l\right)}\left[{h}_{t-1}^{\left(l\right)},{h}_{t}^{\left(l-1\right)}\right]+{b}_{f}^{\left(l\right)})$$

33 $${g}_{o,t}^{(l)}=sigm({W}_{o}^{\left(l\right)}\left[{h}_{t-1}^{\left(l\right)},{h}_{t}^{\left(l-1\right)}\right]+{b}_{o}^{\left(l\right)})$$

34 $$  c_{t}^{{(l)}}  = c_{{t - 1}}^{{(l)}} \, ^\circ g_{{f,t}}^{{(l)}}  + tanh(W_{c}^{{\left( l \right)}} \left[ {h_{{t - 1}}^{{\left( l \right)}} ,h_{t}^{{\left( {l - 1} \right)}} } \right] + b_{c}^{{\left( l \right)}} )\, ^\circ \,g_{{in,t}}^{{(l)}}  $$

35 $${h}_{t}^{(l)}={\mathrm{tanh}(c}_{t}^{(l)})^\circ {g}_{o,t}^{(l)},$$

where ${W}_{*}^{\left(l\right)}$ and ${b}_{*}^{\left(l\right)}$ illustrate the weights and biases of the *l*th LSTM layer, respectively; ${g}_{in,t}^{(l)}$ denotes the input gate; ${g}_{f,t}^{(l)}$ represents the forget gate; ${g}_{o,t}^{(l)}$ is the output gate; ${c}_{t}^{(l)}$ is the memory cell; and ${h}_{t}^{(l)}$ is the output of the *l*th LSTM layer with a given *t*th input.

## Experimental results and analysis

**Figure 6:**
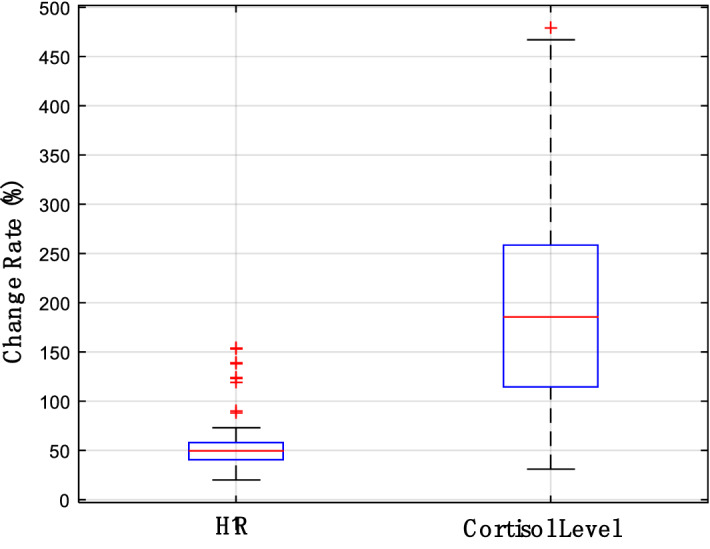
Change ranges of cortisol level and HR under stress conditions.

**Figure 7:**
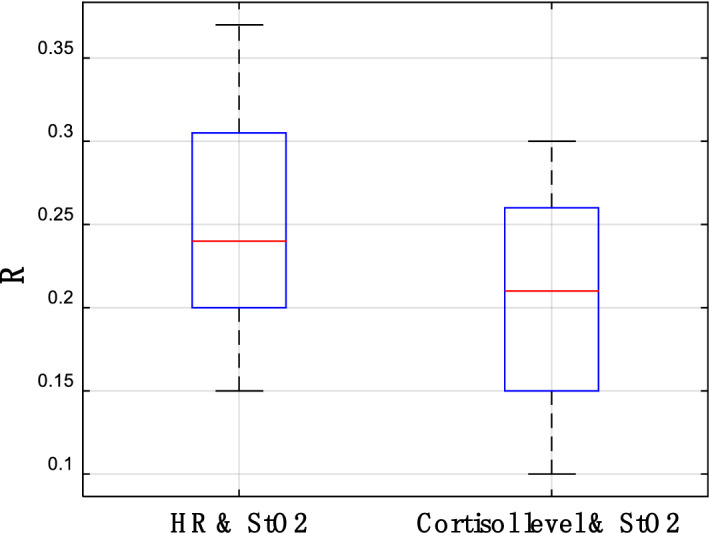
Correlation R value between StO_2_ and ground truth (cortisol level and HR) under stress conditions.

**Figure 8:**
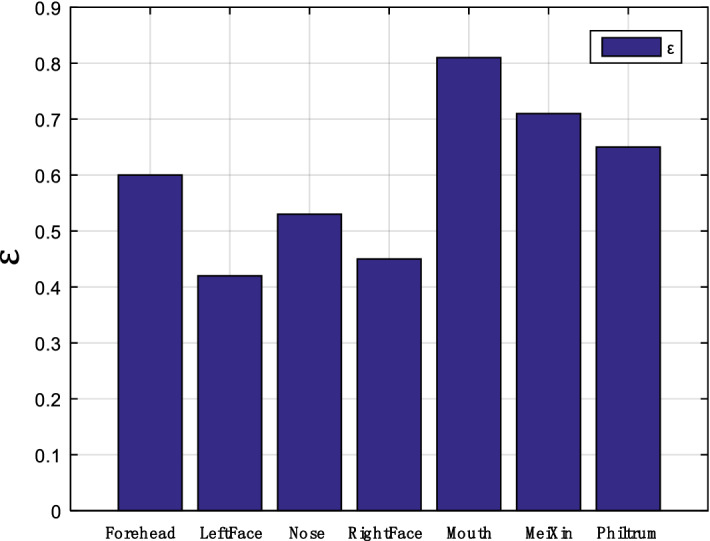
$\upvarepsilon$ value (the ROI selection criterion) obtained by selected ROI.

**Figure 9:**
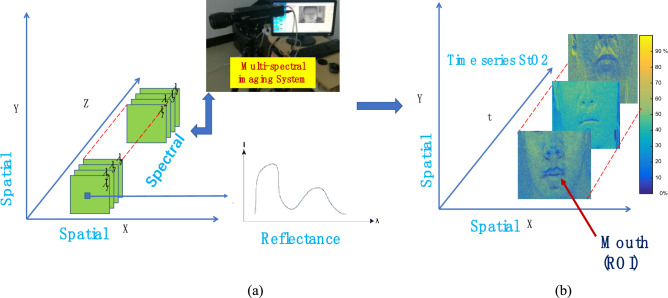
ROI experimental data example. (**a**) Illustration of the experimental setup of the imaging system and the spectral reflectance of relevant pixels. (**b**) StO_2_ signal sample of ROI. As shown in the (**b**), the color bar on the right reflects the percentage of StO_2_.

The experimental data are initially summarized, and the physiological parameters obtained are then used to determine whether the experiment is successful. The change in stress ground truth in the experiment is shown in Fig. 6. From the data results, HR and cortisol content have obtained considerable changes. We compare the ground truth data between baseline and stress status, and Cohen’s *d* indicates a significant practical difference of 1.02, which proves that the stress simulator in this study is feasible. At the same time, this study also attempts to correlate the sequential StO_2_ signal directly with the ground truth (cortisol level and HR). The most anticipated result is that when the ground truth changes, the time series StO_2_ signal also changes, and the two can obtain a high correlation. In this manner, the stress can be directly classified and judged by this correlation; however, unfortunately, it does not occur. The index R value representing the correlation between them is shown in Fig. 7;

Therefore, this study should focus on feature extraction based on deep learning. According to the SC-Net algorithm model, the ROI selection applied to the classification of PS and ES is initially determined. As explained above, the forehead, nose, left face, right face, mouth, meixin (middle eyebrow area), and philtrum are used as the possible ROI. The normalized parameters are taken as the selection criteria. The data results are shown in Fig. 8, and $\upvarepsilon$ value represents the ROI selection criterion. According to the experimental results, the ROI selection parameter of the mouth area obtains the highest value, that is, the area that best meets the ROI selection criteria. Therefore, the mouth is selected as the ROI. Examples of the obtained ROI data are shown in Fig. 9. The experimental setup of the imaging system and the spectral reflectance of relevant pixels are illustrated in Fig. 9a. StO_2_ signal sample of ROI is presented in the Fig. 9b, and the color bar on the right reflects the percentage of StO_2_.

Through further extraction and processing of the ROI signals, the classification features are obtained. The final classification results of PS and ES are shown in Table 1, which is highly encouraging, that is, the accuracy rate of the proposed algorithm exceeds 90%. A one-tailed paired Student’s *t*-test identifies a significant difference (*p* < 0.005) between the feature values for the ES and PS conditions. In addition, Cohen’s *d* indicates a large practical difference of 1.39. This significance suggests that the feature values are favorable indicator for ES and PS classification.

**Table 1:** Confusion matrix of the SC-NET algorithm for final classification result.

	Emotional stress	Physical stress
Emotional stress	96.7%	3.3%
Physical stress	3.3%	96.7%

Furthermore, the proposed algorithm is compared with the machine vision and the artificial intelligent classification algorithms in the field. However, comparing it with public data is impossible, because the proposed algorithms and experiments in this study are based on spectral imaging; thus, the comparisons here are based on the data produced in this study and those of different algorithm models. Furthermore, we also compare the proposed algorithm with the previous stress classification methods in Table 2, such as thermal imaging^83^ and HSI^103^ based stress classification methods. As shown in Table 2, the accuracy of other algorithms is lower than that of the proposed SC-Net algorithm, which reaches the accuracy rate of 96.7%. The proposed algorithm model achieves better accuracy, which shows its robustness.

**Table 2:** Final Accuracy of PS-ES classification obtained by other algorithms in the field.

Methods	Classification rate (%)
KNN	83.6
Decision tree	85.1
HSI based SVM^103^	95.5
Densenet	79.8
Bayes	77.5
EM-CCA^83^	93.3

At the same time, the proposed algorithm is divided into different parts to determine whether different parts of the algorithm (especially input features) can have a positive effect on the whole accuracy. Different feature structures are used as input to the spatial–frequency network. As shown in Table 3, the classification model is divided into the following features: Feature 1 (MSI), Feature 2 (MSI + StO_2_), Feature 3 (MSI + StO_2_ + ROI selection), and Feature 4 (MSI + StO_2_ + ROI selection + MN-VLAD). This setting is designed to demonstrate the influence of the input feature on the results of the algorithm. Different input features are placed into the SC-Net model for PS and ES classification (Table 3). The addition of different parts of process further improves the accuracy of the algorithm. The data processing model shows a positive response to the experimental results. Before the feature extraction, corner point detection and optical flow method are employed to eliminate the effect of the participants’ tremble. When the participant just finished running, the participant is still under PS status. Moreover, the participants have already sat still. Therefore, this PS dataset can be obtained when participant sit still. To this end, this set of PS data and ES data can be put into the SC-NET for testing. The experimental results are tested by t-test, and the results show that no statistical difference exists in the recognition results of different status in the above data. The null hypothesis that the experimental classification results of PS situation (during running and right after running) are different is disproven. Therefore, SC-NET classifies the type of stress instead of simply the activity.

**Table 3:** Comparison of the average accuracy of different input features calculated using the SC-Net model.

Input feature	Accuracy (%)
Feature1	58.7
Feature2	76.9
Feature3	83.5
Feature4	96.7

**Figure 10:**
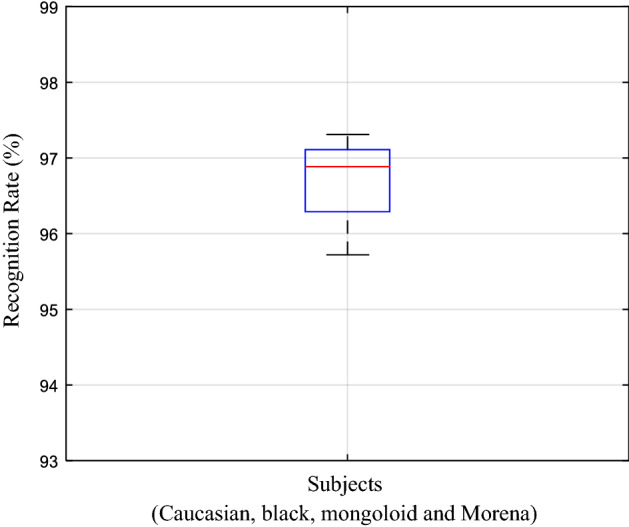
Algorithm accuracy under different skin colors of participants; the composition ratio of each skin color is 25%.

In addition to comparison with other algorithms, the parameters involved in the proposed algorithm are also tested in-depth, including the influence of skin color on the algorithm. To ensure the objectivity of the experiment, the composition ratio of each skin color is 25% (each skin color have 32 participants). The recognition rate results of the algorithm under different skin colors are shown in Fig. 10. Four skin colors (i.e. Caucasian, black, Mongoloid, and Morena) are selected as experimental objects, and the classification and recognition rates of ES and PS under different skin color conditions are illustrated in Fig. 10. From the figure, no obvious difference is observed in classification and recognition rates among different skin colors. The experimental results are tested by t-test, and the results show that no statistical difference exists in the recognition results of different skin colors in the above data. The null hypothesis that the experimental results of these different skin colors (correct rate) are different is disproven (p > 0.6 in all experimental data).

## Discussion and conclusion

In this study, a classification algorithm of ES and PS based on MSI technology and StO_2_ is developed. Previous studies have also proved that StO_2_ is sensitive to stress. However, extracting stress classification signals directly by using StO_2_ somewhere on the face as ROI remains extremely difficult. At the same time, the commonly used HSI system cannot provide real-time facial signals. Therefore, an MSI system is used to obtain the near-real-time StO_2_ signals of the human face under ES and PS. Then, the SC-Net algorithm model based on deep learning is proposed.

After the proper ROI is obtained, the signal is put into the SC-Net to learn the classification signal. The experimental result demonstrates that the accuracy rate exceeds 90%, which proves the feasibility of the algorithm. To verify the effect of each step on the results of the algorithm, the test data are put into different steps, and the accuracy of the algorithm is then verified again. The results are still highly encouraging, which shows that the proposed algorithm steps meet expectations. Further, the proposed algorithm is compared with the machine vision and the artificial intelligent classification algorithms in the field. However, comparing it with public data is impossible, because the proposed algorithms and experiments in this study are based on spectral imaging; thus, the comparisons here are based on the data produced in this study and those of different algorithm models.

To the best of the authors’ knowledge, this study is the first attempt in the field of affective computing to classify PS and ES by using multispectral imaging. Therefore, there is no directly comparable method. But we can make the comparison with the previous stress classification methods, such as hyperspectral imaging^103^ and thermal imaging^83^ based stress classification methods. As with the aforementioned experimental result, the accuracy of other algorithms is lower than that of the proposed SC-Net algorithm, which reaches the accuracy rate of 96.7%. The proposed algorithm model achieves better accuracy, which shows its robustness. A one-tailed paired Student’s *t*-test identifies a significant difference (*p* < 0.005) between the feature values for the ES and PS conditions. In addition, Cohen’s *d* indicates a large practical difference of 1.39. This significance suggests that the feature values are favorable indicator for ES and PS classification. Further, Different input features are placed into the SC-Net model for PS and ES classification. The addition of different parts of process further improves the accuracy of the algorithm. The data processing model shows a positive response to the experimental results. Apart from the recognition accuracy, no obvious difference is observed in classification and recognition rates among different skin colors. The experimental results are tested by t-test, and the results show that no statistical difference exists in the recognition results of different skin colors in the above data. The null hypothesis that the experimental results of these different skin colors (correct rate) are different is disproven (p > 0.6 in all experimental data).

Future work still needs greater efforts, and several methodological limitations must be solved. First, ROI tracking algorithm must be further developed. The ROI of the proposed algorithm is the facial area of the human being. The accuracy of classification algorithms highly depends on the quality of data source. It will drop down if the dataset is blurred or disappeared. Although the data source in this study is spectral images, all tracking algorithms in the RGB image field can essentially be utilized. Therefore, the research and development of the ROI tracking algorithm based on spectral signals will be the focus of future works. Second, the classification characteristics of stress can be further refined. For example, before putting the classification features into the deep learning model, the features can be pre-subdivided according to the characteristics of ES and PS. Therefore, in follow-up works, the proposed algorithm model needs more biomedical knowledge and data to improve. At the same time, the data range can be further expanded, and more participants and experimental results can be inputted into the project to increase reliability.

In short, SC-Net, a classification model of ES and PS based on MSI technology, is presented in this study. The proposed method can effectively explore the differences between ES and PS in spectral and StO_2_ signals, and on the basis of this difference, related experiments and algorithm models are constructed. The experimental results are encouraging, which proves the robustness of the SC-Net algorithm. The proposed algorithm is less affected by external environmental factors, which can lay a good foundation for the application of stress identification and classification. It will have important application prospects in the fields of security and healthcare, which will be continued in future studies.

## Data Availability

The datasets generated during the current study are not publicly available due to the fact that most of the dataset is facial region image. However, the dataset is available from the corresponding author on reasonable request.
